# Guiding Nipple-Areola Complex Reconstruction: Literature Review and Proposal of a New Decision-Making Algorithm

**DOI:** 10.1007/s00266-020-02047-9

**Published:** 2020-11-20

**Authors:** Guido Paolini, Guido Firmani, Francesca Briganti, Michail Sorotos, Fabio Santanelli di Pompeo

**Affiliations:** 1 Faculty of Medicine and Psychology, Plastic Surgery Department, Sapienza University of Rome–Sant’Andrea Hospital, Via di Grottarossa, 1035-1039, 00189 Rome, Italy; 2 Department of Medicine, Surgery and Dentistry “Scuola Medica Salernitana”, PhD School of Translational Medicine of Development and Active Ageing, Università degli Studi di Salerno, Salerno, Italy; 3 Chair of Plastic Surgery, Faculty of Medicine and Psychology, Sapienza University of Rome - Sant’Andrea Hospital, Via di Grottarossa, 1035-1039, 00189 Rome, Italy

**Keywords:** Nipple-areola complex, Nipple-areola reconstruction, Nipple-areola surgery, Breast reconstruction, Breast cancer

## Abstract

**Background:**

Nipple-areola complex reconstruction (NAR) most commonly represents the finishing touch to breast reconstruction (BR). Nipple presence is particularly relevant to the patient’s psyche, beyond any shadow of doubt. Many reconstructive options have been described in time. Surgery is easy, but final result is often disappointing on the long run.

**Methods:**

The goal of this manuscript is to analyze and classify knowledge concerning NAR techniques and the factors that influence success, and then to elaborate a practical evidence-based algorithm. Out of the 3136 available articles as of August 8th, 2020, we selected 172 manuscripts that met inclusion criteria, which we subdivided into 5 main topics of discussion, being the various NAR techniques; patient factors (including patient selection, timing and ideal position); dressings; potential complications and finally, outcomes/patient satisfaction.

**Results:**

We found 92 articles describing NAR techniques, 41 addressing patient factors (out of which 17 discussed patient selection, 14 described ideal NAC location, 10 described appropriate timing), 10 comparing dressings, 7 studying NAR complications, and 22 addressing outcomes and patient satisfaction. We elaborated a comprehensive decision-making algorithm to help narrow down the choice among NAR techniques, and choose the correct strategy according to the various scenarios, and particularly the BR technique and skin envelope.

**Conclusions:**

No single NAR technique provides definitive results, which is why we believe there is no “end-all be-all solution”. NAR must be approached as a case-by-case situation. Furthermore, despite NAR being such a widely discussed topic in scientific literature, we still found a lack of clinical trials to allow for more thorough recommendations to be elaborated.

**Level of Evidence III:**

This journal requires that authors assign a level of evidence to each submission to which Evidence-Based Medicine rankings are applicable. This excludes Review Articles, Book Reviews, and manuscripts that concern Basic Science, Animal Studies, Cadaver Studies, and Experimental Studies. For a full description of these Evidence-Based Medicine ratings, please refer to the Table of Contents or the online Instructions to Authors www.springer.com/00266

## Introduction

Nipple-areola complex reconstruction (NAR) most commonly represents the finishing touch to post-oncologic breast reconstruction (BR) [1–3]. Despite being a minor procedure, it holds major significance to the patients [4], because of its impact on the overall appearance of the breast [5–9]. Scientific literature describes an overabundance of possible NAR techniques. Some of those techniques have emerged in time as valid options that are still popular and currently in use, whereas most others have been abandoned throughout the years. No single technique proved to resist loss of nipple projection, which inevitably occurs over time [4]. That is why final results are often disappointing on the long run.

Interestingly enough, BR is offered to cancer patients using a wide variety of options, ranging from implant-based techniques to autologous-based flaps. Different techniques find different indications according to patient and tumor characteristics. Conversely, NAR is approached according to personal preference alone [10], regardless of the circumstances. For this reason, we decided to perform a review of scientific literature to address the most relevant topics regarding NAR to identify what factors can modify the end-result. An “end-all be-all” solution might not exist [11], which is why the goal of this manuscript is to also describe a decision-making algorithm to offer the most satisfying results in NAR in all different varieties of BR patients.

## Materials and Methods (Fig. 1)

**Fig. 1:**
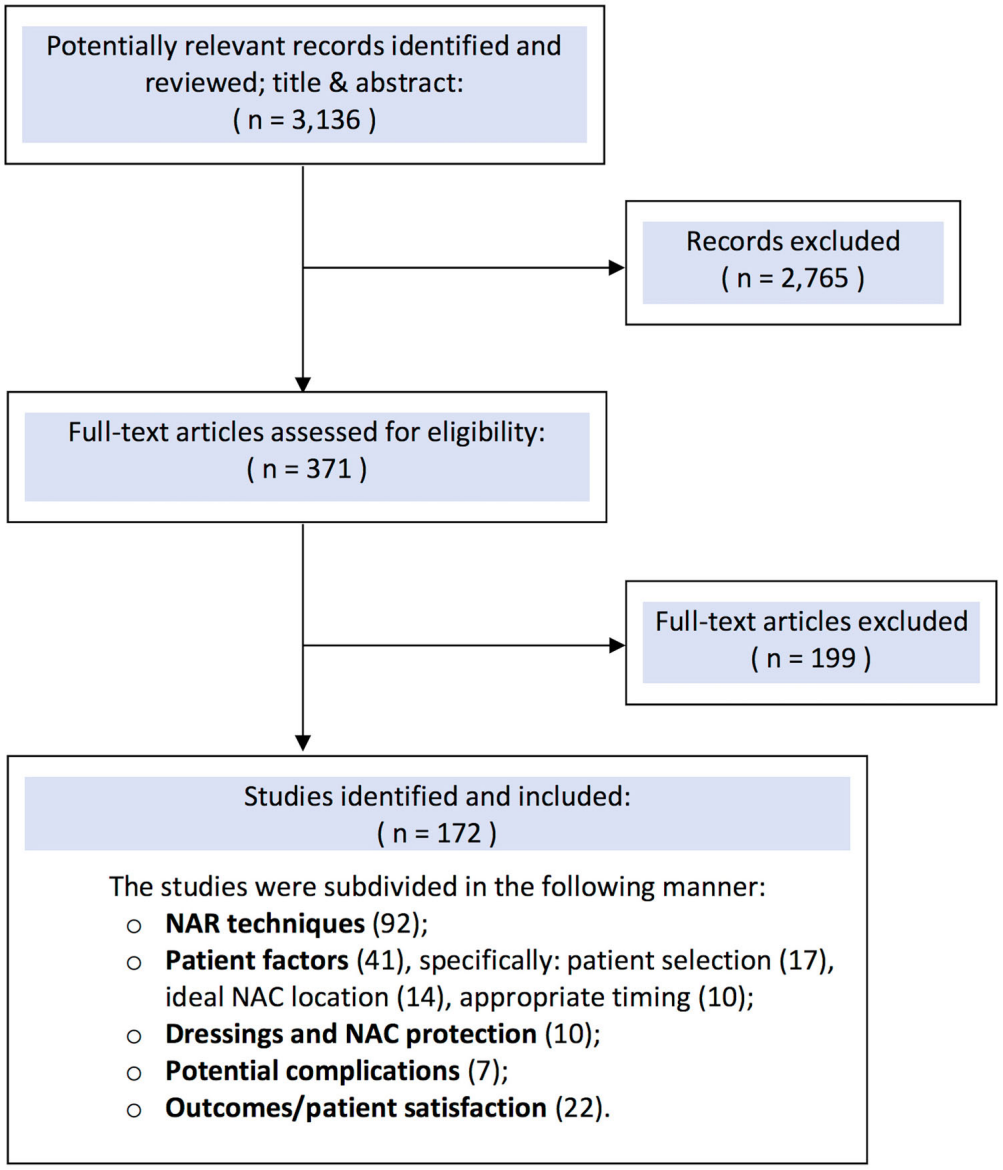
Flow diagram representation of the search strategy with included and excluded articles

We performed a review of scientific literature by searching through the following electronic bibliographic databases: PubMed/MEDLINE, Embase, ScienceDirect, Cochrane Library and Google. We selected all eligible articles written in English and French, and pertaining to human-based topics, by identifying pertinent index terms (Medical Subject Heading [MeSH]) and relevant free-text keywords. The search terms we used were “Nipple-areola complex” OR “Nipple” OR “Breast cancer” AND “Surgery” OR “Reconstruction” OR “Plastic Surgery”. We classified the articles based on five main topics of discussion, being NAR techniques; patient factors, including ideal patient selection, timing and position of the NAC; dressings and protective devices; potential complications; and finally, outcomes/patient satisfaction. Ideal NAR patient selection specifically included Stage I-III breast cancer patients that underwent skin-sparing, skin-reducing or radical mastectomy. Metastatic breast cancer (Stage IV) and breast cancer treated with local treatments, including breast-conserving surgery and nipple-sparing mastectomy, were excluded. Our aim was to identify evidence-based measures to offer optimal NAR results to breast cancer patients, and to elaborate a decision-making algorithm to guide the choice of the techniques. The review search was conducted between February 2020 and August 2020 by G.F. and F.B. Four reviewers (G.P. G.F., F.B., and M.S.) independently reviewed the titles and abstracts yielded by this comprehensive search and subsequently selected articles based on the predetermined inclusion and exclusion criteria. Disagreements were resolved by an additional reviewer (F.S.d.P.) or through consensus-based discussion. Out of the 3136 articles that were available as of August 8th, 2020, we excluded all duplicate articles and articles discussing nipple-sparing mastectomy, BR, nipple inversion, nipple hypertrophy or other benign NAC conditions, selecting a total of 172 articles.

## Results

We found 92 articles describing NAR techniques, 41 addressing patient factors (out of which 17 discussed patient selection, 14 described ideal NAC location, 10 described appropriate timing), 10 comparing dressings, 7 studying NAR complications, and 22 addressing outcomes and patient satisfaction.

### Nipple and Areola Reconstruction Techniques

We found 92 articles that discussed nipple and/or areola reconstruction techniques following mastectomy. There are over 60 counts of unique NAR techniques that have been described in the past 8 decades, the first dating all the way back to 1944 with Adams’ NAC transplantation during a breast reduction procedure [12], and to 1946 with Berson’s attempt at recreating a nipple prominence with a local flap for a breast cancer patient [13]. Although complete description of all existing NAR techniques is beyond the scope of this manuscript, we will provide an accurate account of all existing categories described in the literature.

**Fig. 2:**
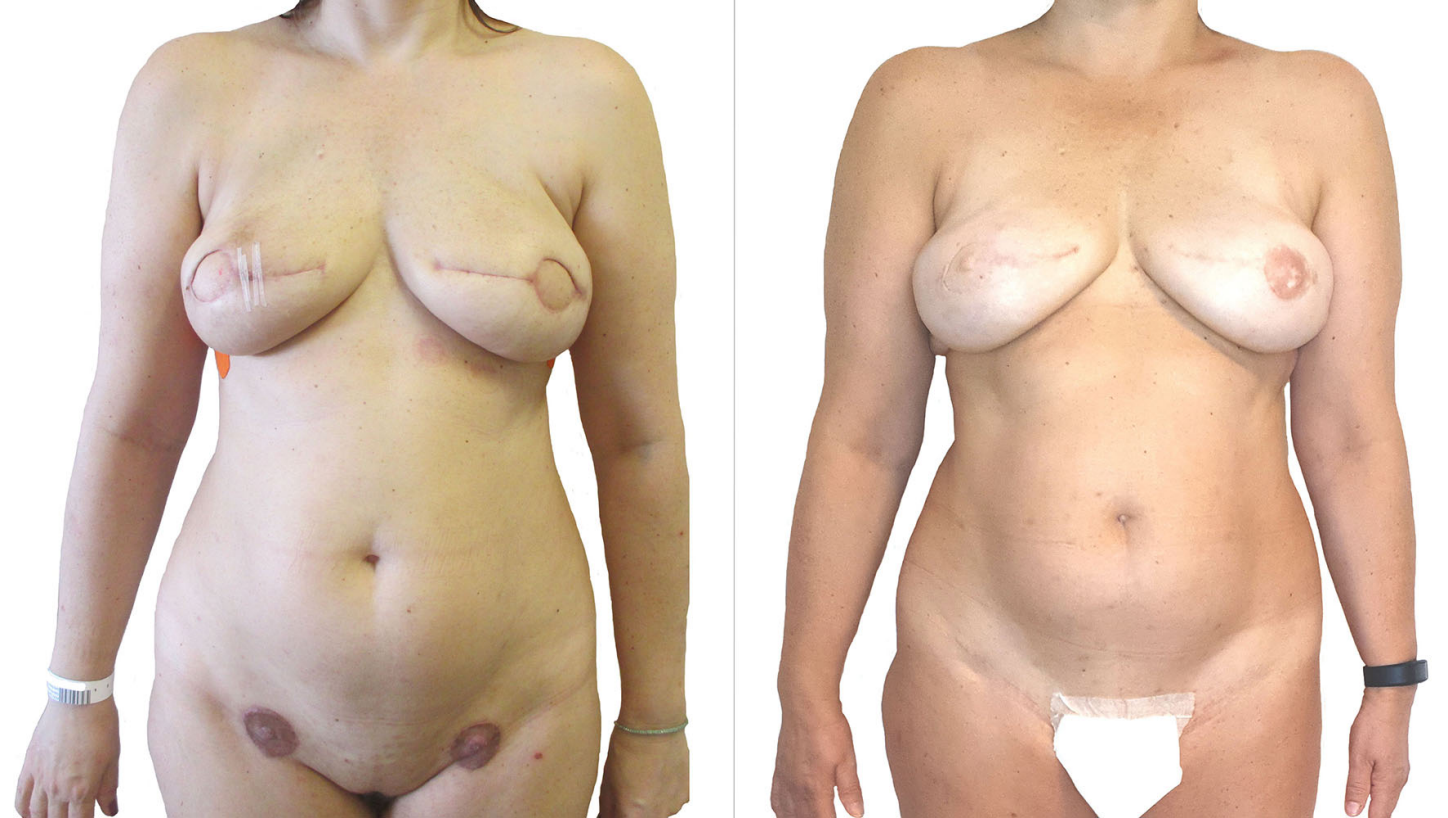
Patient at 3 weeks follow-up after bilateral skin-sparing mastectomy and immediate breast reconstruction with fat-augmented latissimus dorsi (FALD) flap for bilateral infiltrating ductal carcinoma (*Left*). Areolas were initially replaced with LD skin and NAC were banked in the inguinal crease. Right NAC was later discarded for oncologic reasons, and the new NAC was reconstructed with C-V flap 3 months later. Left NAC was transplanted 3 months later from the inguinal region to the breast mound, showing mild discoloration and loss of nipple projection. Patient refused any additional procedures to reconstruct the right areola and improve the left and returned to follow-up 2 years later (*Right*)

Available techniques for areola reconstruction include skin grafting, banking and replantation, and tattooing. Skin grafting is an adjunct technique to other NAR procedures that recreate the nipple. It originally started in 1949 with Adams et al. [14], and was later modified by Brent et al. in 1977 [15]. It consists in harvesting a circular-shaped full-thickness skin graft and placing it around the neo-nipple. Donor site is usually selected according to the original color of the areola: if light pink, the graft can be harvested from the oral mucosa. If darker, suitable donor sites include the labia minora and majora from the groin [16, 17], the buttock [18] or the upper thigh [19], because skin harvested from these areas supposedly tends to hyperpigment [6]. Some propose harvesting the outer rim of the contralateral areola, which can be useful in patients with large NAC who would simultaneously benefit from an areolar reduction [20]. If no donor site is available, Seaman proposes using an acellular dermal matrix (Alloderm) as an onlay graft [21]. NAC banking, first introduced by Millard in 1971 [22], consists in removing NAC from the breast and replanting them in the groin area to allow time for the BR to heal. After extemporaneous/definitive examination, the spared NAC is reattached on the reconstructed breast to complete NAR (Fig 2). Some authors describe cryopreservation techniques for replantation after definitive histologic exam report [23, 24]. With the latter approach, replantation occurs after 5.8 months on average. If cancer cells are diagnosed in the meantime, during histological examination, the specimens are discarded. Intradermal tattooing (or dermopigmentation) for areolar reconstruction was first introduced by Bunchman et al. in 1974 [25] and popularized by Becker in 1986 [26]. It usually lasts 20 to 30 minutes per NAC in an outpatient setting. It might require topically applied anesthetic according to the patient’s degree of sensation [11]. The operator can offer a wide variety of choices in terms of pigment selection according to the contralateral areola’s color in unilateral NAR [11], or to the patient’s skin tone in bilateral NAR. Nevertheless, dermopigmentation is just an adjunct to other techniques, as it only provides the illusion of a texture without a projection.

**Fig. 3:**
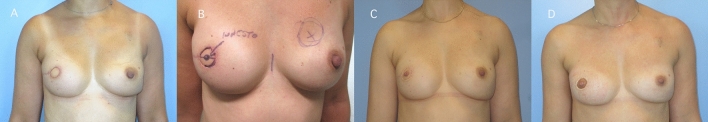
Patient at 12-month follow-up for left nipple-sparing mastectomy and right skin-sparing mastectomy, with immediate breast reconstruction using a bilateral DIEP flap (**a**). She underwent right nipple reconstruction using Lossing’s S-flap and a fat grafting session of the left breast during the same setting under general anesthesia 6 months later (**b**). Patient returned 4 months later for a single dermopigmentation session for areolar reconstruction (**c**) and 5 more months later for follow-up (**d**)

Available techniques for nipple reconstruction: nipple sharing, local flaps, augmentation grafting using autologous or heterologous materials, prosthesis or 3D tattoos. Millard et al. first introduced nipple sharing in 1972 [27]. Its use remains popular when contralateral nipple has a projection of over 1 cm [28]. There are two main techniques: longitudinal splitting and decapitation [29]. For the latter approach, at least 50% of the donor nipple’s original height must be left intact to ensure adequate residual projection and sensation. The composite nipple is sutured on a de-epithelialized recipient area using interrupted sutures. Most authors advocate for the use of stitches to close the tip of the donor nipple [29], though Haslik et al. favor second intention healing, which occurs in 10 days on average and provides an equally acceptable scar and sensibility [30]. Local flaps represent the most commonly described technique for nipple reconstruction [6, 31]. They have significantly evolved in time, and to date, over 60 techniques have been described. They can generally be classified into three categories according to the principle upon which they are raised [5]: centrally based flaps, subdermal pedicled flaps and pull-out/purse-string flaps. Centrally based flaps include Berson’s “pseudo-nipple” reconstruction, introduced in 1946 which consisted in using 3 triangular split-thickness skin flaps, Barton’s blunted Maltese cross in 1982, Little’s quadrapod flap in 1983, and Cohen’s pinwheel flap in 1986. Subdermal pedicle flaps represent the most common category of local flaps. They can be based on a single pedicle, including Snyder’s V-Y advancement flap in 1972, Little’s skate flap in 1987, Anton and Hartrampf’s star flap in 1991, Jones and Bostwick’s C-V flap in 1994, and Guerra’s arrow flap in 2003; they can be bipedicled, such as Kroll’s double-opposing-tab flap in 1989, Cronin’s S-flap in 1988 and Lossing’s modified S-flap in 1998 (Fig 3); or they can even be based on a triple pedicle such as Krogsgaard’s triple flap design in 2019 [32]. Pull-out/purse-string flaps include Hallock’s H flap in 1993, Eng’s Bell flap in 1996, Hamori’s Top-hat Flap in 1998, Shestak’s double opposing peri-areolar flap in 2007 and the Hammond Flap in 2007. Most local flaps are considered “dermal-fat” flaps because they consist in raising full-thickness skin flaps that reach subcutaneous tissues. However, some authors have described strictly dermal flaps that can be safely used in implant-based BR patients, even with a history of radiotherapy (RT), such as Rem et al.’s inferiorly pedicled dermal flap [33]. Augmentation grafting can be approached as a primary NAR. However, it generally represents a secondary/revision measure to improve structural support and enhance the projection of a flattened neo-nipple [11]. It requires the use of either autologous or heterologous materials [7]. Autologous tissues include dermis [34, 35], adipose tissue [36], cartilage (from the ribs or the outer ear) and mucosa (from the gums of the oral cavity or the rima ani at the coccygeal level) [1]. Costal rib cartilage can be harvested during free flap-based BR procedures, when internal mammary vessels are selected as recipient site [37, 38]. In these cases, internal mammary vessel dissection requires the resection of a small portion of costal cartilage which can be banked and reused for augmentation grafting of the reconstructed nipple. Used heterologous materials can be classified as synthetic or allogenic [7]. Synthetic materials are foreign bodies which serve as internal implants and include silicone gel or rods [39], hyaluronic acid, calcium hydroxylapatite (Radiesse^TM^)[40], artificial bone substance (Ceratite^TM^) [41, 42] and polytetrafluoroethylene [43]. Acellular dermal matrices (AlloDerm^TM^, GCDerm, SureDerm) and the Biologic Collagen Cylinder [10] are classified as allogenic materials [11, 44]. They use decellularized nipple scaffolds that support recellularization with the host’s own cells [45, 46]. When patients refuse additional surgeries or cannot safely undergo them, they may be eligible for 3D tattooing, which unlike traditional dermopigmentation, includes shadings and details, creating an optical illusion to compensate for the lack of a nipple [9, 47, 48]. Another solution is external prosthetics, which represent an inexpensive and completely atraumatic solution^8^ but not a reconstructive option.

### Patient Factors

We found 41 articles discussing patient factors related to NAR. Out of those, 17 articles discussed patient selection for a successful NAR approach. The ideal candidate to NAR procedures has aesthetically pleasant breast mounds with adequately vascularized soft tissues [3]. Certain risk factors can negatively impact wound healing and make NAR more difficult to achieve, including smoking and RT [49, 50], that increase risk for necrosis of the neo-nipple [6, 50, 51]. The chosen BR technique is also relevant when planning NAR. Breast-implant reconstruction patients usually have thin, contracted and poorly vascularized soft tissues that are more at risk of reconstructive failures. Irradiated patients with breast implants have a breast skin envelope that is thinned even further. Raising dermal or subcutaneous skin flaps for NAR in these cases may be contraindicated [3, 26], and nipple sharing is considered the safest technique [29, 37, 52, 53]. However, Yong Hong et al. routinely perform NAR on implant-based breast mounds using local flaps by carefully selecting patients [54]. They only perform the procedure when: (a) tissue expansion exceeds the mastectomy weight; (b) the expanded skin is sufficiently elastic, and (c) sufficiently thick. Nevertheless, Chao [55] described a “delay procedure” to safely implement local flaps, even in irradiated patients with breast implants. It consists of three steps: (1) the incision of the skin and dermis following the shape of the flap; (2) a delay of 2.5 weeks on average, to ensure viability of the skin flaps, then the raising of the flaps; (3) and finally areola tattooing several months later. Bernard advocates for autologous fat grafting sessions along the markings of a chosen flap design, to increase subdermal fat thickness. He then delays the procedure to stabilize the result and then raises the flap to complete NAR [33]. In patients with previous scarring or who previously underwent NAR and require revision, Riccio et al. advise using the V-Y flap or Kroll’s double-opposing flap to incorporate mastectomy scars, thereby decreasing new scar formation and improving aesthetic results. Other local flaps that incorporate scars include Cronin and Lossing’s S-flap [56].

We found 14 articles that studied the ideal position for successful neo-nipple placement in NAR planning. Authors describe NAR placement according to the position on the breast mound, along with shape, size and projection of the nipple [2, 3]. In unilateral reconstructions, the contralateral nipple serves as a template. In bilateral reconstructions, there is no general rule and the surgeon must select the most suitable and aesthetically pleasant position of the neo-nipple using anatomical landmarks [45], all the while keeping in mind the preference of the patient [1]. Lewin et al. suggested that the ideal NAC is located in the middle of the breast, vertically and slightly lateral to the midpoint horizontally [57], while Laschuk et al. proposed the “rule of thirds”, according to which the areola should represent just under one third of the base width, and the nipple should represent about one third of the areolar diameter [58]. Nevertheless, surgeons will usually place the NAC on the point of highest projection on the breast mound [30], which often corresponds to a sternal notch-to-nipple distance of 19–21 cm and a nipple-to-inframammary fold distance of 7–8 cm [59]. In implant-based BR, Young Hong advises adding 0.5 cm to the sternal notch-to-nipple distance when replacing a tissue expander with a definitive implant, since capsular contracture is a common occurrence that should be taken into account [55]. He discusses adding 1.0 cm instead when planning to perform contralateral balancing procedures (mastopexy, breast reduction), to compensate for the downward shift of the contralateral nipple that will occur over time. In regard to ideal NAC measurements, some authors recommend a 4–7 mm nipple diameter, a height of > 1 cm and an areolar diameter of 4.2–4.5 cm^4^. A review of 600 reconstructed NAC reported an average diameter of 1.3 cm, a height of 0.9 cm and a 1:3 nipple-to-areola ratio [60, 61]. Furthermore, when NAR is planned using local flaps, preoperative markings usually incorporate an overcorrection by 25–50% of the desired result [62, 63], since flattening is inevitable [3, 64].

We found 10 articles that discussed the most appropriate timing when planning for NAR. There are reports in the literature of surgeons performing NAR immediately (at the time of BR) [55, 65, 66]. However, there is a general agreement in favoring delayed nipple reconstruction, as a final step at least 3–6 months after BR surgery [3], and 2–4 months after large revisions [11, 67], once the breast mound is stable, to avoid a potentially asymmetric nipple placement if placed too early [5]. Surgeons should also wait for their patients to conclude their adjuvant chemotherapy/RT regimens before planning NAR. In fact, irradiating a newly reconstructed NAC increases the risk of poor wound healing and flattening. Furthermore, if patient has undergone implant-based BR, RT increases the risk of capsular contraction which might dislocate the reconstructed nipple causing malposition [3]. In regard to areolar reconstruction, dermopigmentation is delayed by at least 6 to 12 weeks from the nipple reconstruction, to allow proper healing [1]. Some authors choose to reconstruct the nipple with flaps immediately after having tattooed the surrounding skin [68], stating that this approach supposedly guarantees an even uptake of pigment, even along the scars. However, most authors do not recommend this approach, since the additional trauma of flap dissection to the pigmented skin might reduce vascularity of the flap [1].

### Dressings and Nac Protection

We found 10 articles addressing reconstructed nipple protection and dressings. The main concern in the postoperative course consists in protecting the nipple, and ensuring a correct compression over the skin graft (if the latter approach was used to reconstruct the areola) while still avoiding direct pressure to the reconstructed nipple for the first 7 days [69]. Many authors describe various types of dressings. In 1995, Monteiro advised anchoring the graft with eight symmetrically placed 4–0 silk stitches, then placing a petrolatum gauze with a central cut over the nipple, covering the entire graft [70]. He then proposed placing the rubber stopper of a 60-mL syringe over the nipple, with cotton balls packed around it, then folding the petrolatum gauze over the stopper and the dressing, and tying the sutures. In 1997, Papay et al. proposed a similar technique but using the non-adherent xeroform gauze on the skin graft and four-to-five layers of DuoDerm, both with a central hole for the nipple projection [71]. Liew et al. described in 2001 a technique using a Tielle hydropolymer dressing with a central hole to accommodate the nipple and then placing an extra layer of gauze and microfoam tape as padding [72]. Saravolac et al. described the use of a moldable thermoplastic thick foam with a hole in the middle as a nipple shield [73]. Lim et al. found in 2010 that Gamgee tissue pads could be used as a dressing to improve the perfusion of the reconstructed nipple [74], while Salgarello in 2008 [75] and Weissman in 2010 [76] investigated the use of silicone shields as a way to better protect nipple from compression. Sircar [77] and Staruch [78] recommended placing a Cartella eye-shield or similar eye protectors, over the NAR dressings, while Rosing et al. tested in 2010 the use of the Asteame Nipple Guard [79]. In general, excessive compression of the nipple should be avoided to prevent microcirculation impairment [75].

### Complications to Nar

We found 7 articles discussing NAR complications. There are no significant differences in terms of complication rates between the various local flap techniques [6]. The most commonly reported complications include partial and total nipple necrosis (1–29%), which was found to be more prominent in irradiated patients [11], infection (0.9–16%) and complete loss of projection which warranted a reoperation (2–24%). The most severe reported complication was the loss of breast implants due to their exposure [80]. Other complications include donor-site morbidity. Nipple sharing technique can cause major concerns in terms of loss of sensibility in the healthy nipple [81]. Autologous augmentation grafting can cause donor site morbidity (i.e., ribs or outer ear for cartilage, gums of the oral cavity or the rima ani for mucosa) [1]. A meta-analysis revealed that augmentation grafting with synthetic materials has a higher rate of complications [63], especially Ceratite [42]. On the other hand, allogenic materials provide a projection comparable to that of autologous tissues [7], with no donor-site morbidity and lower overall complication rate than that of both autologous and synthetic grafts [45, 82]. However, they represent the most expensive alternative out of the three [45]. Dermopigmentation gained popularity for being the least invasive areolar reconstruction technique, and for not carrying significant donor-site morbidity. It has a low risk of complications, which mainly consist in local infections, wound dehiscence if performed too soon after local flaps [83], and rare allergic and photosensitive reactions caused by pigments [1].

### Patient Satisfaction and Outcomes

Patients who undergo NAR are overall more satisfied with their BR than those who do not [3, 84]. However, patient satisfaction related to NAR results mostly depends on the maintenance of nipple projection and color matching [29, 85, 86]. Conversely, NAR dissatisfaction usually stems from a lack of long-term nipple projection and color mismatch, as well as unappealing shape and nipple malposition [3, 85]. We found 22 articles discussing NAR satisfaction and outcomes. Jabor et al. found that the longer the interval between breast mound reconstruction and NAR, the less satisfied the patient [85]. Surprisingly, they found that breast mound type, RT and NAR technique had no influence on patient satisfaction [85]. The most common outcome to NAR is loss of projection, caused by the flattening that often occurs over time [4], regardless of which technique was used for the breast mound (autologous vs implant-based) [11, 68]. This is due to retraction forces of the surrounding and underlying tissues and scar contraction which reduces blood flow [5]. However, it is more evident after tissue expansion (where the dermis and subcutaneous tissues are thinned) and following adjuvant RT [45]. Nevertheless, it is less pronounced on implant-only breast mounds [87], probably because the implant provides a more solid foundation. For autologous BR, abdominally based flaps and gracilis flaps provide a relatively thin dermis, which makes them more prone to tissue contraction and nipple projection loss [8, 35, 45, 88] in comparison with latissimus dorsi and gluteal flaps, which provide a thicker dermis [45]. Local flaps lose projection with a range from 17% to over 75% [11, 81], and an average of 25–50% [1]. Current trends in local flaps reject centrally based patterns because they fail at creating long-lasting projections. They are characterized by centrifugal retraction forces which cause increased tissue contraction, leading to shrinkage of the flap [45]. NAR has moved toward local flaps with simple designs and subdermal pedicles [5]. The more complicated the local flap design, the more the scarring, which reduces blood supply to the skin flaps and increases tissue contraction thus causing shrinkage and loss of projection [5]. Subdermal pedicle flaps exert considerably lower retraction forces and redistribute them more evenly, which translates into a longer-lasting projection. In regard to areola reconstruction, achieving the correct color match constitutes the main challenge [5]. Areolar grafting has lost popularity because of patient dissatisfaction with pigmentation, which is hard to predict, and has higher donor site morbidity [1]. NAC banking and replantation has also been abandoned or limited for safety concerns, as several cases of cancer cell infiltration in inguinal lymph nodes have been reported [89, 90]. Furthermore, banking frequently damages the NAC causing loss of projection and pigmentation, especially when using cryopreservation [23]. The authors that still advocate its use suggest applying it only when breast cancers are remote from the native NAC [3]. Dermopigmentation’s main drawback is fading [11], which occurs over time in as many as 60% of patients [5, 11, 69]. However, this is manageable with touch-up sessions to apply new pigment [11]. Additionally, areolar tattooing requires a learning curve to apply the pigment at the correct depth, being the upper and mid-papillary dermis [5]. If too superficial, the pigment lies in the epidermis that sheds at 30-days turnover. If too deep, lymphatic vessels drain the pigment. In both cases, the tattoo fades causing a dissatisfying result [1, 5]. This is why some authors advise for the use of a professional tattoo artist, which can, however, be costly.

### The Sant’Andrea University Hospital (SAUH) Decision-Making Algorithm for NAR (Fig. 4 a, b)

**Fig. 4:**
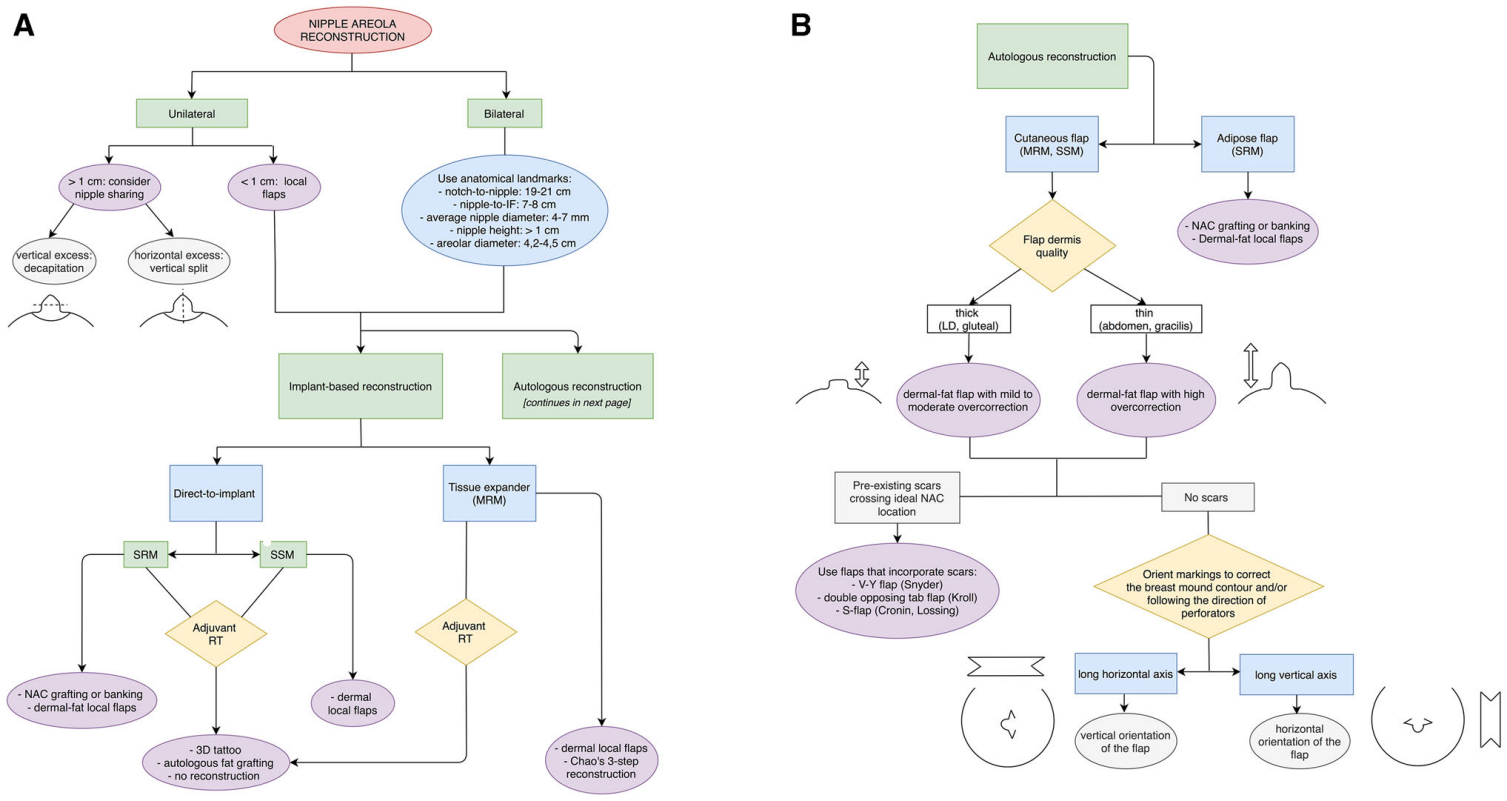
The Sant’Andrea University Hospital (SAUH) decision-making algorithm for nipple-areola reconstruction, with (a) general recommendations and specific recommendations for implant-based BR, and (b) specific recommendations for autologous-based BR

**Fig. 5:**
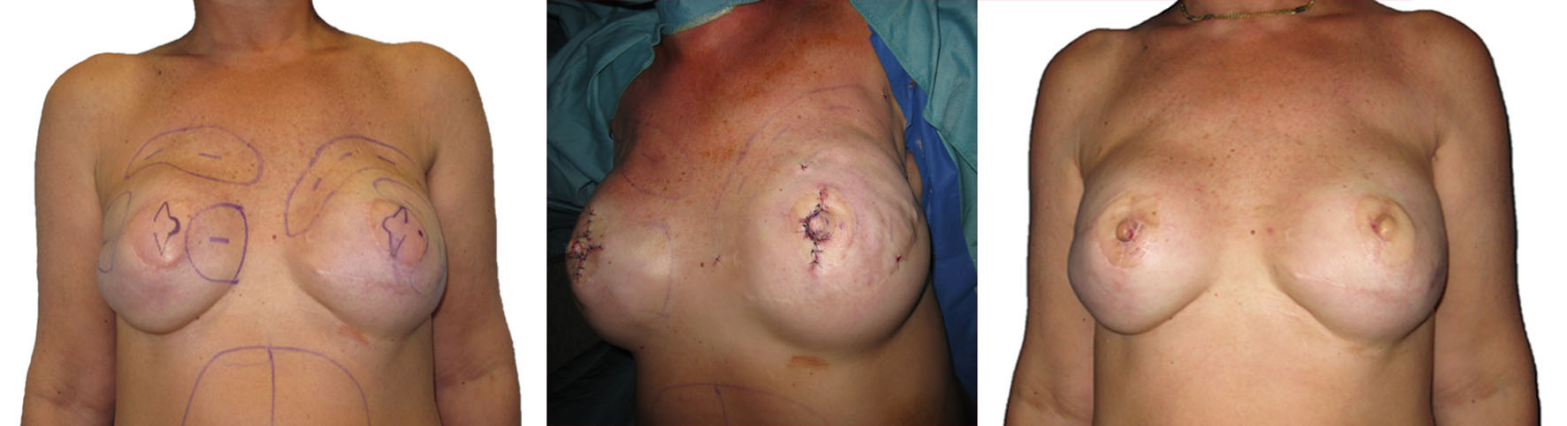
Patient at 6-month follow-up after secondary breast reconstruction with implant-enhanced latissimus dorsi flap (*Left*) after failed implant reconstruction of bilateral skin-sparing mastectomy. Patient underwent a fat grafting session and bilateral C-V flap for nipple reconstruction during the same operative setting (*Center*). Preoperative markings were oriented in accordance with the NAR algorithm to reduce breast mound asymmetries. Patient at 6-month follow-up following NAR (*Right*)

This practical algorithm has been elaborated following an accurate review of 172 manuscripts discussing various aspects of NAR. It describes 6 categories of possible NAR approaches: 1) nipple sharing, 2) NAC banking/replantation, 3) “dermal-fat” flaps, 4) strictly dermal flaps, 5) tattooing and 6) no reconstruction at all. The starting point should take into consideration laterality. When nipple reconstruction is unilateral, the contralateral side should be used as template. When the unaffected nipple has a projection > 1 cm, consider using nipple sharing, otherwise use local flaps. The most commonly used flaps include the skate, star, C-V and arrow flaps [44]. Conversely, when reconstruction is bilateral, previously mentioned anatomical landmarks should be applied to appropriately locate the NAC. Mastectomy type and BR technique can influence end-results of NAR and therefore assess the quality of the skin envelope before choosing an option. As plastic surgeons, we designed the algorithm putting an emphasis on the used BR technique, separating implant and autologous–based procedures, and later characteristics of the skin envelope. In patients that underwent staged expander/implant reconstruction, dermis and subcutaneous tissues may be thinned. In these cases, nipple sharing might represent a safe option. If said option is not possible and usual flaps do not guarantee an uneventful outcome, Chao et al.’s delayed 3-step technique for local flaps is feasible. Alternatively, 3D tattooing can provide satisfactory results. In Direct-To-Implant BR, quality of dermis and subcutaneous tissues depends on patient characteristics and aggressiveness of the skin-sparing mastectomy (SSM). In cases of SSM with an average skin thickness, local flaps may be attempted, but dermal pedicle-based techniques should be favored to reduce the risk of implant exposure [33]. In the case of skin-reducing mastectomies (SRM) in which NAC can be spared, NAC banking and immediate [91] delayed replantation may be used when oncological concerns are ruled out. In the case of adjuvant RT, skin might become fibrotic and thin. In these cases, autologous fat grafting can be used before planning local flaps [33]; otherwise, NAR should be avoided, opting for a 3D tattoo instead. In autologous-based BR, in patients undergoing SRM with NAC preservation, NAC may be immediately reimplanted over the de-epithelialized dermis of the underlying buried autologous flap, to avoid the risks of delayed healing of the mastectomy skin flaps due to poor terminal vascularization. NAC can otherwise be banked, performing replantation at a later stage, once flap has settled in the correct position (Fig 2). In patients that underwent SSM or radical mastectomy, NAC is preferably reconstructed directly onto autologous flap skin due to improved vascularity. In these cases, the quality of the flap dermis can affect NAR. Latissimus dorsi and gluteal flaps provide a thick dermis, making local flaps less pliable but requiring only mild-to-moderate overcorrection when planning NAR. On the other hand, abdominal and gracilis flaps provide a thinner dermis which make them more pliable and easily moldable but require a higher degree of overcorrection. In either case, if a pre-existing scar is located on the ideal position for the NAC, it would be most suitable to select a local flap technique that incorporates the scar, such as Snyder’s V-Y flap, Kroll’s double-opposing tab flap or Cronin’s S-flap. If no scars are present, local flap markings can be oriented in a specific manner to correct breast mound contour asymmetries. If the transverse diameter is too long, markings may be oriented vertically to shorten the mound length. If the vertical axis is excessive, markings may be oriented horizontally to reduce mound width [35] (Fig 5). Another element worth taking into consideration in autologous-based BR is to orient local flap markings away from scars to take advantage of the flap’s vascular autonomization, or along the direction of the perforators, in the vicinity of the perforasome “hot spot”, where blood supply is increased [92] and thus might reduce the risk of projection loss.

## Discussion

Ingenious surgical innovations have led to the creation of an impressive number of NAR techniques that all aim at providing natural-looking projections with adequate shape and size. Most described techniques have been relegated to the past [1, 64]. However, some have grown to become widely accepted and are still popular today. Nevertheless, all of the currently available options still fail at providing consistent results [7, 93], when indifferently used with different reconstructive modalities. This problem may be related to the fact that previous authors failed at pointing out the real variables affecting NAR success, mainly addressing the efficacy of their preferred reconstructing technique more than the substratum on which it was realized (reconstructive technique and consequent skin envelope).

We found just two algorithms in the literature that described recommendations on how to approach NAR. Lee et al. introduced the Kyungpook National University Hospital (KNUH) algorithm in 2019 [94]. It is based on the experience that the authors developed following a study on 21 patients who underwent implant-based BR and NAR using C-V or Hammond flap and that were followed-up for 12 months to assess results. This algorithm provides recommendations on when to perform NAR immediately and when to delay them, and clarifies how to manage potential complications. However, it is limited by three elements: firstly the small number of patients recruited in the study, secondly the lack of diversity in NAR techniques, since only 2 local flaps were tested, disregarding other safer alternatives in implant-based BR such as nipple sharing [29], and thirdly they recruited only implant-based reconstructions, ignoring autologous ones. Jian et al. described a NAC reconstruction algorithm in 2018 that was based on scientific literature. It addresses NAR in both implant and autologous-based BR [95]. The main limitation to their algorithm is the indeterminateness of the recommendations which we found when addressing NAR in autologous BR. The authors select NAR techniques according to laterality and contralateral nipple size alone. We believe that the type of breast mound reconstruction and consequent skin envelope calls for different nipple reconstructive techniques and affects the success of the chosen one. We found that several factors can impact the choice of the techniques and their success: the type of skin envelope, the type of autologous flap used (since it can affect the thickness of the dermis and therefore the need for overcorrecting local flap markings), the presence of scarring across NAC location and the direction of perforators (to maximize local flap vascularity) and finally the presence of breast contour asymmetries (to plan local flap markings accordingly, in order to correct them). Therefore, all mentioned factors must be taken into consideration.

Despite being technically simple, NAR are still surgical procedures that are not exempt from possible complications that can tarnish the end-result. Nipple reconstruction is considered a failure when > 80% of the starting projection is lost in time [64]. In these cases, revision procedures are available for secondary NAR reconstruction. Some authors advocate the use of augmentation grafting to enhance the projection [33], while others repeat local flaps [96]. However, added scarring reduces blood flow and increases the risk of projection loss^5^. In those cases, we recommend the use of flaps that incorporate previous scars [57].

Additionally, we found very few accounts of randomized or controlled clinical trials attempting to compare specific NAR techniques between each other [4]. Shestak et al. demonstrated the superiority of the skate and the star flap to the bell flap [64]. Rubino et al. proved that the arrow flap was superior to a modification of the star flap [97], and Alfano et al. proved that the skate flap retained nipple projection for longer than the star flap [98]. Kroll compared the double-opposing tab flap to the star flap, finding the first to retain a slightly better nipple projection after 2 years of follow-up (2.42 vs 1.97 mm), particularly over breast mounds reconstructed with Transverse Rectus Abdominis Muscle (TRAM) flaps [99]. Pizzonia et al. compared their purse-string and immediate fat grafting augmentation technique with traditional techniques in patients with thin breast skin envelope, obtaining worse projection (5 ± 2 mm vs 8 ± 2 mm) but lower projection loss [100].

NAR is widely performed and represents a pivotal factor in patient satisfaction after BR [4]. So much so that scientific literature brims with research papers on the subject. And yet, examples of clinical trials available are few and far between. This hinders the possibility of presenting more specific recommendations that are based on solid and undeniable scientific evidence. Trials evaluating the efficacy and results of a single technique in different clinical settings or different techniques in a single clinical scenario are welcome.

## Conclusion

We are still currently far from the ideal NAC reconstruction method, and with there still not being an “end-all be-all” solution, we recommend choosing an option on a case-by-case situation, using our proposed decision-making algorithm to narrow down the available options. We also found a dire need for more evidence-based research on the subject of NAR to make our recommendations more thorough in the future.
